# Targeting Bacterial Adenylate Kinase mRNA with a Chimeric Antisense Oligonucleotide for Rational Antibacterial Drug Development

**DOI:** 10.3390/molecules30163425

**Published:** 2025-08-20

**Authors:** Lozena A. Otcheva, Martina Traykovska, Robert Penchovsky

**Affiliations:** Laboratory of Synthetic Biology and Bioinformatics, Faculty of Biology, Sofia University “Saint Kliment Ohridski”, 8 Dragan Tzankov Blvd., 1164 Sofia, Bulgaria

**Keywords:** antisense oligonucleotide, multi-drug resistant bacteria, antibacterial drug development, *Staphylococcus aureus*, antibiotics, antibiotic resistance

## Abstract

Multi-drug resistance in human bacterial pathogens has become a significant challenge for global healthcare this century, mainly due to the widespread misuse of antibiotics worldwide. As a result, millions of people have been affected by multi-drug-resistant bacterial infections. The antibiotic development pipelines cannot cope with the need to produce new antibiotics. Therefore, more productive antibiotic development methods must be invented. This paper presents an entirely rational approach for antibacterial drug discovery based on chimeric antisense oligonucleotide targeting (ASO) of the adenylate kinase mRNA in *Staphylococcus aureus*. The ASO is delivered into the bacteria via the cell-penetrating oligopeptide pVEC. The pVEC-ASO1 exhibits a bactericidal effect against *Staphylococcus aureus*, with a 50% minimal inhibitory concentration of 500 nM. The pVEC-ASO1 has a 98% survivability rate at the same concentration on cell lines. These findings strongly suggest that this chimeric ASO is a promising antibacterial drug candidate. Moreover, this is the fifth bacterial mRNA we have successfully targeted with pVEC-ASOs, providing further evidence for the efficiency of our approach. In contrast to the previous four targets, riboswitches residing in the 5′-untranslated region, we target the coding part of mRNA found in bacteria. That suggests that our approach may have much broader therapeutic applications.

## 1. Introduction

Antibiotics have been successfully used to cure bacterial infections and as prophylactic agents in humans since the early 1940s and later in animals. However, due to their misuse, antibiotic resistance (AR) has emerged and become a global problem because it is responsible for the failure of many antibiotic therapies [1]. AR has challenged healthcare since the introduction of the first antibiotics in clinical practice due to the emergence of many antibiotic-resistant (ABR) bacterial pathogens [2]. ABR bacterial pathogens can emerge via the natural selection of mutations and horizontal transfer of genetic information, which are natural features of bacteria. Many causes that have led to AR are due to inappropriate medical therapy, overuse and abuse of antibiotics, secondary consumption of antibiotics in food from animals treated with antibiotics, and other factors. Antibiotic stewardship applied worldwide may help reduce the spread of antibiotic resistance (AR). Still, this cannot eliminate the emergence of AR because AR is part of the very nature of bacteria. Therefore, we must develop new antibiotics against ABR bacteria, particularly against multi-drug-resistant (MDR) bacterial pathogens, such as MDR *Staphylococcus aureus*.

MDR, including methicillin-resistant *S. aureus*, emerged following the introduction of penicillin, tetracyclines, and methicillin during the 1960s, and unfortunately it persists to this day [3]. *S. aureus* is a commensal Gram-positive bacterium that usually inhabits the respiratory tract, female reproductive tract, skin, and soft tissues. It is also an opportunistic pathogen that primarily causes skin infections, resulting in moderate to severe wounds in adults and severe wounds in children and individuals with compromised immune systems. The scars can be caused by cellulitis [4,5,6], folliculitis [7], skin abscesses [8,9], carbuncles [10], impetigo [11,12], atopic dermatitis [13], sinusitis [14], osteoarthritis [15], or device-acquired infections [11]. It can also cause lethal infections in immune-compromised patients, such as meningitis [12,13,14], pneumonia [15,16,17], sepsis [18,19,20,21], endocarditis [22,23,24], and bacteremia [22,25,26,27,28].

*S. aureus* infections can be acquired in the community or at a hospital [29,30]. It is believed that 20% to 30% of the human population are long-term carriers of *S. aureus*. It is also assumed that approximately 500,000 hospital patients annually in the USA contract a staphylococcal infection, 80% of which are caused by *S. aureus*. Around 50,000 deaths in the USA are linked to staphylococcal infections each year. Without antibiotic treatment, *S. aureus* bacteremia has a fatality rate of around 80%. With antibiotic treatment, case fatality rates range between 15% and 50%. Mortality of staphylococcal infections that are unsuccessfully treated depends on the age and health status of the patient, as well as the AR of the *S. aureus* strain. It compromises social and individual well-being and leads to serious economic concerns. Therefore, we must develop new antibiotics against *S. aureus* to prevent a post-antibiotic apocalypse.

However, many pharmaceutical companies refrain from developing new antibiotics because their application is limited in terms of time and the number of patients, and therefore is not particularly profitable compared to anticancer, antihypertension, anticholesterol, and diabetes drugs, which are taken daily for a prolonged period. Thus, novel approaches for antibacterial drug development (ABDD) that are more efficient than those based on high-throughput screening of small molecules need to be developed.

Recently, we have developed a practical approach for ABDD based on chimeric antisense oligonucleotides (ASOs) conjugated to the cell-penetrating oligopeptide pVEC, targeting the riboswitch region of mRNAs in bacteria [31]. Our entirely rational approach includes targeting selection and ASO design. It has been experimentally proven in four instances, probing pVEC-ASOs against SAM-I [32], TPP [33], FMN [34], and glmS [35] riboswitches, which are found in the 5′-untranslated region (5′-UTR) of mRNAs in many human pathogenic bacteria [36]. This approach has been proven very efficient in saving time and money with 100% accuracy in selecting targets and designing ASOs as antibacterial agents [37].

The research presented here extends our approach to engineering ASO-based antibacterial agents, which we refer to as asobiotics, that target non-riboswitch mRNA, such as adenylate kinase. Adenylate kinase (ADK) catalyzes the conversion of adenine nucleotides, including adenosine monophosphate (AMP), adenosine diphosphate (ADP), and adenine phosphoribosyltransferase (APT), thereby regulating cell homeostasis and playing an essential role in cellular processes. ADK is a widespread enzyme found not only in bacteria but also in humans. Thus, we have selected a target region in bacterial mRNA not found in human ADK mRNA. Therefore, we provide evidence that our approach is not limited to targeting riboswitch mRNA but to any RNA.

## 2. Results

### 2.1. Assessing mRNA of Adenylate Kinase as a Target for Antibacterial Drug Development

**Figure 1:**
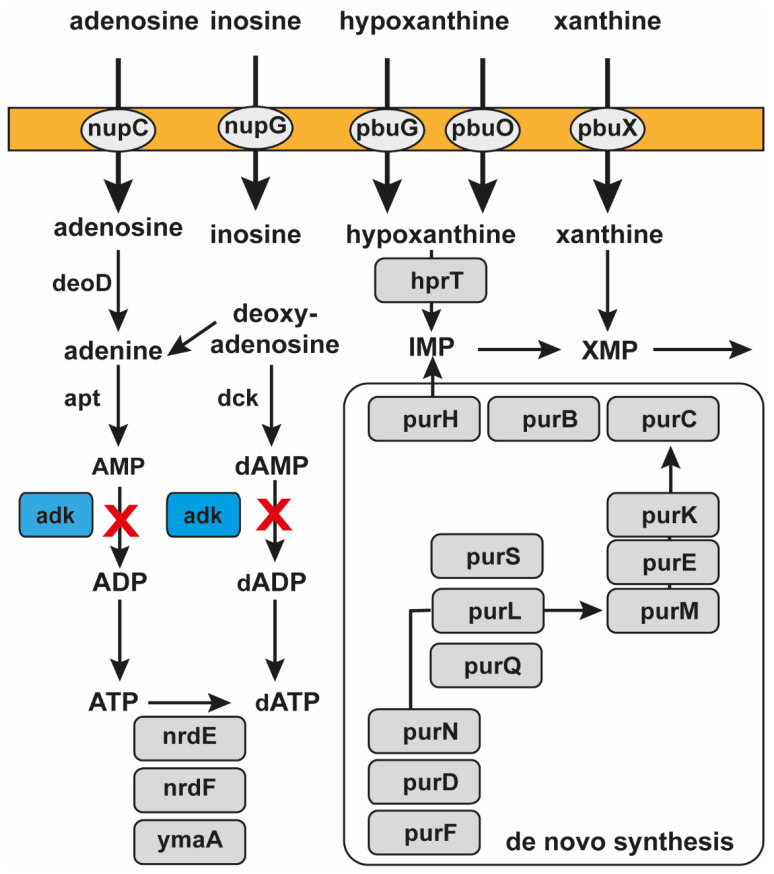
Metabolic pathways involving ADK. “X” (in red) indicates the pathways blocked by pVEC-ASO1 by targeting ADK mRNA in bacteria. There are two pathways blocked by pVEC-ASO1 due to its specific binding to the mRNA of ADK. The first blocked pathway is the conversion of adenosine through adenine and AMP to ADP. The second pathway involves the synthesis of deoxyadenosine through dAMP to dADP. The hybridized region of pVEC-ASO1 and ADK mRNA is recognized and hydrolyzed by RNase H. Consequently, the synthesis of ATP by phosphorylation of ADP, essential for cell survival, is prevented.

Biosynthetic pathways of ATP, ADP, and AMP are interconnected (Figure 1). All these metabolites are essential because the cell cannot survive without their synthesis. Adenosine can be transported into the cell by nucleoside permease (NupC) and transformed into adenine by purine nucleoside phosphorylase (DeoD). Adenine is then converted into AMP by adenine phosphoribosyltransferase (APRT). The interconversion of AMP to ADP is catalyzed by ADK (Figure 1). Deoxyadenosine can be phosphorylated to form deoxyadenosine monophosphate (dAMP) by deoxycytidine kinase (dCK) (Figure 1). Analogously, the interconversion of dAMP to deoxyadenosine diphosphate (dADP) is also catalyzed by ADK (Figure 1).

ADK (EC 2.7.4.3) is a phosphotransferase catalyzing the interconversion of ATP, ADP, and AMP. The reversible biochemical reaction is as follows: ATP + AMP ⇔ 2 ADP. ADK is a universal enzyme found in prokaryotes and eukaryotes. ADKs are vital in cellular energy metabolism and nucleic acid synthesis in all organisms. Thus, ADKs play a critical role in cellular energy homeostasis by constantly monitoring phosphate nucleotide levels in eukaryotes. To date, nine ADK enzyme isoforms have been identified in humans. Therefore, finding a target sequence of ADK mRNA that is present only in bacteria but not in humans is essential. When the expression of bacterial ADK is blocked, the further syntheses of ATP, dATP, ADP, and dADP are disturbed, and the bacteria will perish (Figure 1).

**Figure 2:**
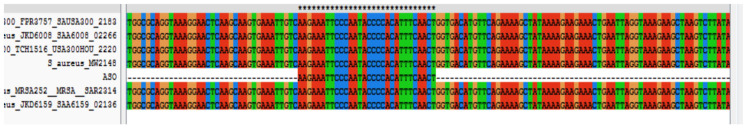
Multiple sequence alignment of the chosen target sequence with ADK mRNAs of several bacterial strains of *S. aureus.* The conserved region of the ADK mRNAs is aligned with the sequences of six *S. aureus* strains. The Clustal X software 2.1 established the identical target sequences. Asterisks indicate identical nucleotides in all aligned sequences.

**Figure 3:**
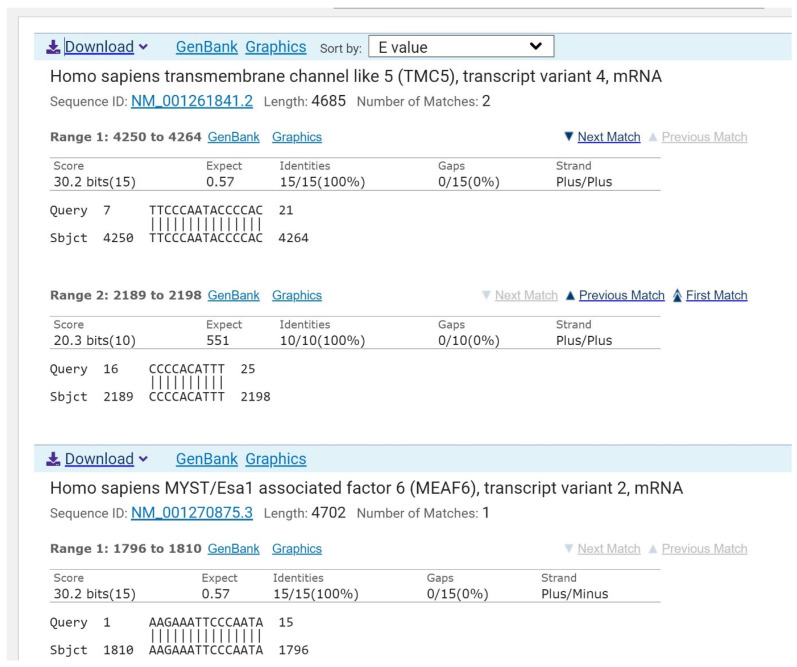
NCBI GenBank alignment results. A BLAST search of the bacterial ADK sequence against the human genome showed no significant hits. Three short, fragmented alignments were found. From the 4685th nucleotide of the human TMC5 mRNA, a total of 25 nucleotides were matched to the ADK sequence, while the third alignment showed a similarity of 15 nucleotides to the MEAF6 mRNA.

The sequence 5′-AAGAAAUUCCCAAUACCCCACAUUU (target_1) is found in the ADK mRNA of 100 bacteria from the genus *Staphylococcus*, including in pathogenic bacteria *S. aureus* NCTC 8325, *S. aureus* N315, *S. aureus* DSM 20231, *S. aureus* sister species *S. simiae*, and *Clostridium scatologenes* ATCC 25775 (Figure 2). The exact sequence of bacterial ADK is not found in humans (Figure 3). This sequence of ADK mRNA can be targeted in pathogenic MDR bacteria via ASO. Thus, according to our criteria, ADK mRNA is a suitable target for designing antibacterial drugs such as asobiotics.

### 2.2. Bacterial Growth Inhibition by Targeting ADK mRNA with pVEC-ASO1

ASO-based therapeutics consist of reverse-complementary modified ASO that hybridize with targeted RNA sequences of interest, forming a DNA/RNA hybrid. If the ASO contains no chemical modifications at all or PS modifications only in the middle of its sequence, ribonuclease (RNase) H recognizes the hybrid region formed and cleaves targeted RNA under multi-turnover conditions. As a result, targeted RNA is degraded. Cell-penetrating peptides (CPPs), such as pVEC, can be employed as carriers of therapeutic molecules, including antisense oligonucleotides (ASOs) [38,39,40].

We have designed pVEC-ASO1 to be reverse-complementary to the target_1 sequence, which is part of ADK mRNA (Figure 2). Phosphorothioate modifications (PS modifications) used in both ASOs in this study are necessary to preserve the ASO from nucleases capable of degrading the ASOs [41]. Phosphorothioate modifications also allow RNase H-mediated hydrolysis of the complementary RNA strand [42]. Phosphorothioate ASOs were also likely to bind proteins, forming disulfide bonds [41]. Therefore, we applied 2′-*O*-methyl modifications in both ASOs described to reduce the protein binding of ASOs to cellular proteins, rendering the ASOs less toxic to mammalian cells [43]. 2′-*O*-methyl-modified ASOs had a higher affinity to RNA targets than their respective 2′-deoxy analogs [44]. Both PS and 2′-*O*-methyl modifications enhance efficacy and specificity at higher concentrations of ASO compared to non-modified DNA [45,46].

**Figure 4:**
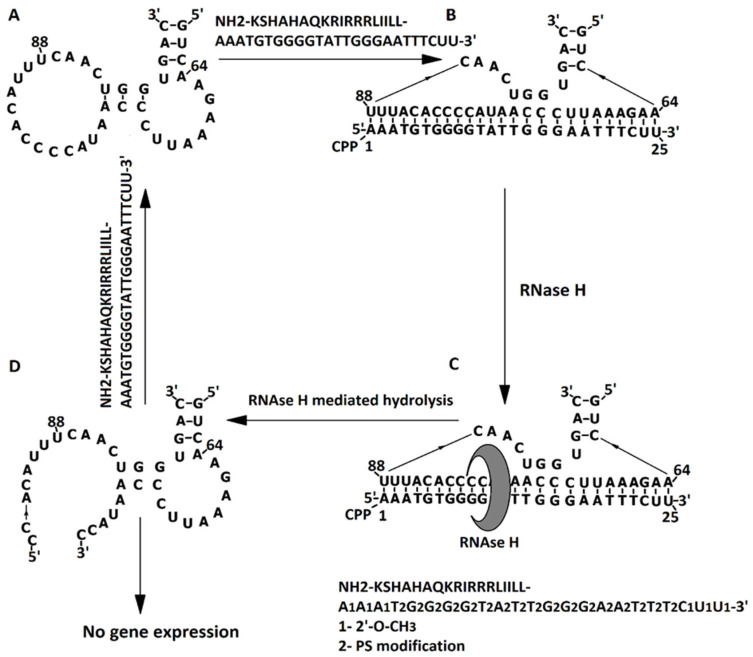
Specific binding of pVEC-ASO1 to ADK mRNA in *S. aureus*. (**A**) The portion of ADK RNA containing the region specifically binding pVEC-ASO1; (**B**) pVEC-ASO1 binds ADK RNA antisense from nucleotide 64 to nucleotide 88. (**C**) The resulting hybrid double-stranded section is recognized by RNase H; (**D**) RNase H mediates the hydrolysis of RNA with the consequent lack of gene expression. pVEC-ASO1 is released after the hydrolysis and is ready to bind to the RNA of other bacteria.

pVEC-ASO1 and pVEC-ASO2 penetrate the cell membrane of *S. aureus* thanks to the cell-penetrating peptide (CPP) pVEC, attached by its carboxyl terminus to the 5′-end of both ASOs. The N-terminus of pVEC is hydrophobic, enabling it to penetrate the lipid bilayer of the cell membrane. Once inside the bacterial cell, the engineered pVEC-ASO1 binds to the ADK mRNA, complementary from nucleotide 64 to nucleotide 88 (Figure 4A,B). The RNase H recognizes the formed hybrid section. This endonuclease enzyme catalyzes the hydrolytic cleavage of RNA (Figure 4C). As a result of the catalytic activity of RNase H, the RNA chain is cleaved, pVEC-ASO1 is released, and it is ready to bind another complementary RNA chain in another bacterial cell (Figure 4D).

**Figure 5:**
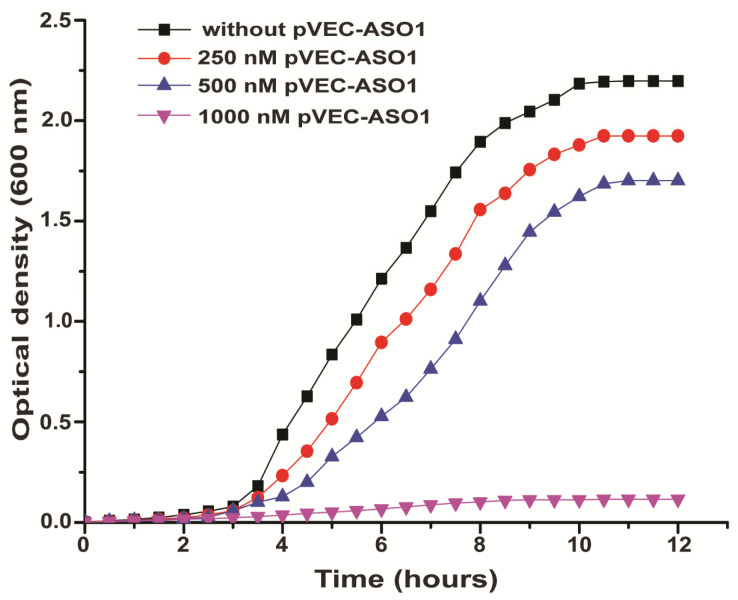
Growth of *S. aureus* according to OD at 600 nm. Bacterial growth in the absence of pVEC-ASO1 (control sample given in black with squares) and in the presence of 250 nM (given in red with circles), 500 nM (given in blue with triangles, peaks pointed upwards), and 1000 nM (given in fuchsia with triangles, peaks pointed downwards) of pVEC-ASO1; bacterial growth in the absence of pVEC-ASO2 (control sample given in black with squares) and in the presence of 1000 nM (given in red with circles) of pVEC-ASO2.

The growth curve of a control sample of *S. aureus* without pVEC-ASO1 is depicted in black with squares. It had an OD of 2.5 after 7 h. Samples with *S. aureus* treated with 250 nM pVEC-ASO1 and 500 nM pVEC-ASO1 (growth curves shown in blue with triangles, peaks pointed upwards, and red with circles, respectively) almost doubled the values of optical density measured every 30 min, as expected due to the binary division of bacterial cells. The concentration of 250 nM pVEC-ASO1 caused no significant decrease in bacterial growth of *S. aureus* compared to the control sample (Figure 5). Growth of *S. aureus* in samples treated with 500 nM pVEC-ASO1 resulted in half the growth of *S. aureus* in the control sample till 5.5 h. All tree growth curves cited reached a plateau phase after 9 h. For samples treated with 1000 nM pVEC-ASO1, a significant inhibition of bacterial growth of *S. aureus* was observed compared to the control sample, indicating a probable bactericidal effect. The OD remained lower than 0.25 after 3 h.

**Figure 6:**
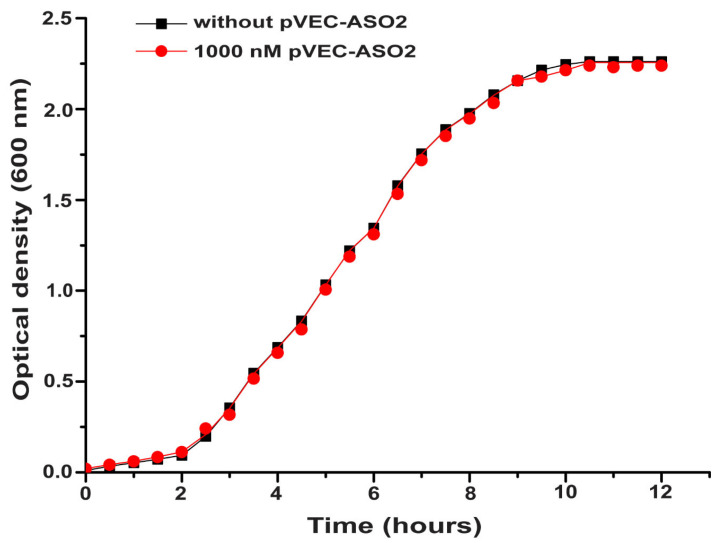
Bacterial growth in the absence of pVEC (control sample given in black with squares) and in the presence of 1000 nM (shown in red with circles) of pVEC. The best bactericidal effect was observed for pVEC-ASO1 at a concentration of 1000 nM due to specific binding. For pVEC-ASO2, as expected, neither bacteriostatic nor bactericidal effect was observed due to the absence of specific binding. pVEC in itself demonstrated no inhibitory effect on bacterial growth.

No decrease in bacterial growth of *S. aureus* in the samples treated with 1000 nM pVEC-ASO2 (a nonspecific binding ASO) compared to the control sample was observed, as expected (Figure 6). The control sample with *S. aureus* only (growth curve shown in black with squares) and the sample with *S. aureus* treated with 1000 nM pVEC-ASO2 (growth curve shown in red with circles), analogously to Figure 5, almost doubled values of OD measured every 30 min, as expected, due to the binary division of bacterial cells. According to the growth curves, a plateau phase for both samples was reached after 9 h at an OD of 2.1 and remained constant over the next 3 h (Figure 6).

No decrease in bacterial growth of *S. aureus* was observed in samples treated with 1000 nM pVEC compared to the control sample (Figure 6). The control sample with *S. aureus* only (growth curve shown in black with squares) and the sample with *S. aureus* treated with pVEC (growth curve shown in red with circles), as in Figure 5, showed almost doubled values of OD measured every 30 min, as expected due to the binary division of bacterial cells. Both samples reached a plateau phase after 9 h at 2.1 OD and remained constant over the next 3 h.

**Figure 7:**
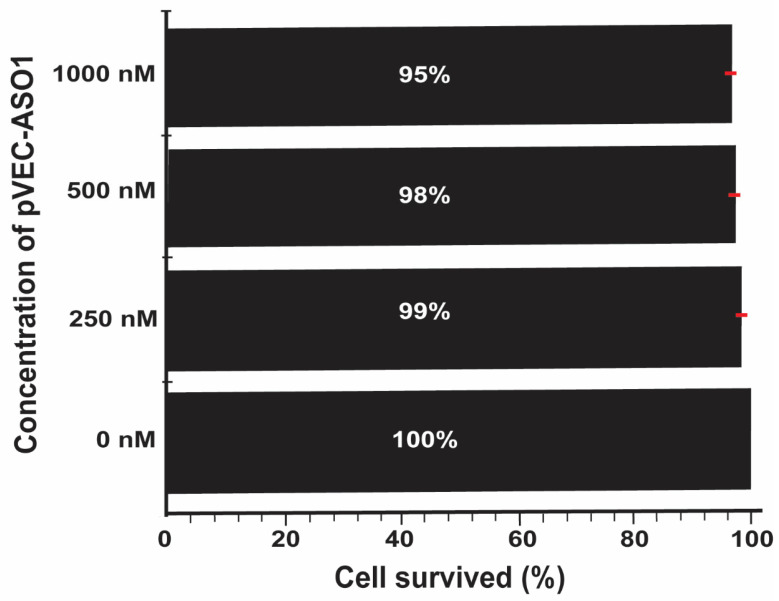
Testing the toxicity level of ASOs in a human cell culture of the non-small lung cancer A549. At the highest concentration of pVEC-ASO-1 used, 1000 nM, the cell survival rate was 95%. At a concentration of 500 nM, the survival rate was 98%, while at a concentration of 250 nM, the survival rate was 99% for pVEC-ASO-1. A 100% survival rate was evident in the sample lacking pVEC-ASO-1.

To test the potential toxicity of pVEC-ASO-1 to mammalian cells, the human non-small cell lung cancer A549 cell line was incubated at varying concentrations of pVEC-ASO-1. The results signified a low toxic effect of the chimeric ASO on the human cells. The survival rate of the cells was recorded at 95% for the highest concentration of 1000 nM of pVEC-ASO-1 and 99% for the lowest concentration of 250 nM. The control sample, which lacked the pVEC-ASO-1, exhibited 100% cell survival (Figure 7).

## 3. Discussion

To date, ASOs are administered to patients and laboratory animals by topical application [16,47] or injection [48,49,50], modified-release tablets [51], There have been studies comparing the oral, intravenous, intraperitoneal, and subcutaneous means of ASO delivery [51] or examining the use of liposomes, artificial virus capsids, cationic acrylate nanoparticles, and protamine-based nanoparticle preparations as carriers of ASOs to determine the most effective delivery method. We can deliver our chimeric ASO via atopic, intravenous, intraperitoneal, and subcutaneous routes.

Targeting bacterial ADK mRNA by pVEC-ASO1 has proven to be a suitable approach for ABDD. The pVEC-ASO1 binds a specific sequence found in the mRNA of adenylate kinase in *S. aureus.* The targeted mRNA sequence is present in bacteria from the genus *Staphylococcus* and other pathogens such as *C. scatologenes*.

In our research, we successfully inhibited the growth of *S. aureus* using the chimeric pVEC-ASOs, ensuring minimal toxicity effects in mammalian cells. This success indicates that our methodology is comprehensive and can be applied to any target RNA in the development of various drug candidates. Combining bioinformatics and genomic analyses, we designed antisense oligonucleotides (ASOs) that specifically target the ADK mRNA of several *S. aureus* strains, thereby avoiding the targeting of mRNA sequences in human cells. Moreover, this is the fifth bacterial mRNA we have successfully targeted with pVEC-ASOs for bacterial growth inhibition [37].

The pVEC-ASO1 exhibits a bactericidal effect against *S. aureus*, with a 50% minimal inhibitory concentration (MIC_50_) of 500 nM. The pVEC-ASO1 has a 98% vitality rate at the same concentration on cell lines. These data suggest that chimeric pVEC-ASO1 is a promising candidate for an antibacterial drug. Unlike the previous four targets, which reside in the 5′-UTR, we targeted the coding part of mRNA found in bacteria. Thus, our approach has much broader therapeutic applications.

## 4. Materials and Methods

### 4.1. Materials

The *S. aureus* strain ATCC 25923 was purchased from the German Collection of Microorganisms and Cell Cultures (DSMZ) GmbH, Braunschweig, Germany. For the storage of *S. aureus,* glycerol cultures were prepared monthly by transferring 500 µL of overnight culture and 500 µL of 60% glycerol and then stored at −80 °C. *S. aureus* colonies are kept fresh by striking an inoculum, a single colony, on a 1.5% agar dissolved in LB broth medium. LB broth containing 1% tryptone produced by Oxoid, 0.5% yeast extract produced by Oxoid, and 1% NaCl in a 50 mL flask was incubated. A total of 100 µL of the culture was transferred to a 1 mL cuvette using a micropipette (Orange Scientific, Braine-l’Alleud, Belgium).

The chimeric ASOs were purchased from GeneLink, Elmsford, NY, USA (Table 1). The manufacturer purified the ASOs via denaturing polyacrylamide gel electrophoresis (dPAGE). They have two chemical modifications and are attached to pVEC. Phosphorothioate (PS) modifications are used in the middle part of the ASOs, while at the two termini, 2′-*O*-methyl modifications are employed. In the case of pVEC-ASO1, the PS modifications are made in nucleotides from 4 to 22, while 2′-*O*-methyl modifications are used in nucleotides from 1 to 3 and in the last three nucleotides. For pVEC-ASO2, the PS modifications are applied in nucleotides from 4 to 8, while 2′-*O*-methyl modifications are used in the first and the last two nucleotides. The 2′-*O*-methyl modifications in both pVEC-ASOs reduce the nonspecific binding of ASOs to cellular proteins, rendering the ASOs less toxic to mammals [43].

### 4.2. Bioinformatics Databases and Software Used

The KEGG pathway collection (https://www.genome.jp/kegg/pathway.html, accessed on 11 June 2025) was used to gain information on metabolic pathways targeted in bacteria. GenBank, a public database available on the NCBI web server (https://www.ncbi.nlm.nih.gov/, accessed on accessed on 11 June 2025), and RSwitch databases (https://penchovsky.atwebpages.com/applications.php?page=58, accessed on 11 June 2025) [52] were used to obtain information on the targeted RNA sequence. Nucleotide BLAST 2.0, a program available on the NCBI web server (https://www.ncbi.nlm.nih.gov/, accessed on 11 June 2025), was used to compare the sequences of interest. The RNAfold web server (http://rna.tbi.univie.ac.at/cgi-bin/RNAWebSuite/RNAfold.cgi, accessed on 11 June 2025) was used to predict the secondary structure of the target gene ADK. The ClustalX software 2.1 (http://www.clustal.org/clustal2/, accessed on 11 June 2025) was used for multiple sequence alignments.

### 4.3. In Vitro Experiments with Bacteria and Human Cell Cultures

An inoculum of a single colony was incubated at 37 °C with shaking in 20 mL of liquid LB medium overnight until it reached an optical density (OD) of 0.7, as measured by a spectrophotometer (Ultrospec 1000, Pharmacia Biotech, Piscataway, NJ, USA) at a wavelength of 600 nm. Two consecutive dilutions of 1:10 of the overnight culture with liquid LB medium were performed, resulting in a final dilution of 1:100.

Bacterial samples were treated with 250 nM, 500 nM, and 1000 nM pVEC-ASO1, as well as without it, to the same fixed final dilution in a total volume of 100 µL. Bacterial samples were treated with 1 µM pVEC-ASO2 in a volume of 100 µL. Bacteria were treated with 1000 nM pVEC at the same volume. All samples contain the same quantity of bacterial dilution. The samples were incubated on a thermostat shaker at 37 °C. Each experiment was performed in triplicate over 12 h, with OD measurements taken every half hour using a spectrophotometer (Ultrospec 1000, Pharmacia Biotech) at a wavelength of 600 nm.

The MTT experiments for the toxicity of pVEC-ASO1 in non-small lung cancer A549 cells were conducted as previously described [53].

## 5. Conclusions

As a strategy for developing novel antibacterial medications, our ASO-based rational approach holds promise for applications in both prokaryotes and eukaryotes due to its ability to inhibit mRNA expression in both types of cells. We can design ASOs that recognize a particular target without nonspecific hybridization to other RNAs by employing a rational design. By cutting costs and time, this strategy can hasten the development of novel antibiotics. We also made a rational selection of mRNA targets for our pVEC-ASOs. The enzyme ADK is crucial for maintaining cellular energy balance. Herein, we provide strong evidence that the growth of Gram-positive bacteria, such as *S. aureus*, can be inhibited by targeting ADK mRNA with pVEC-ASO1. Because of that, the MIC_50_ of our ASO is comparable to that of small molecular weight antibiotics. Our ASO exhibited negligible toxicity to human cell lines.

All experiments followed good laboratory practice guidelines and current bioethical considerations.

## Figures and Tables

**Table 1:** ASO and pVEC sequences. All 5′-terminus amino-modified ASOs were attached to the COOH-terminus of CPP pVEC via the formation of a peptide bond. The ASOs were purchased from GeneLink Ltd. and purified with denaturing PAGE. The “_1_” indicates 2′-*O*-methyl D-ribose modifications at the 2′-position in the D-ribose ring, which are applied in the middle part of the ASO. The “_2_” indicates phosphate (PS) modifications, where one non-bridging O atom in the phosphate group is replaced with an S atom.

Name	Sequence	Target
pVEC-ASO1	5′-A_1_A_1_A_1_T_2_G_2_T_2_G_2_G_2_G_2_G_2_T_2_A_2_T_2_T_2_G_2_G_2_G_2_A_2_A_2_T_2_T_2_T_2_C_1_U_1_U_1_	ADK
pVEC-ASO2	5′-U_1_A_1_C_2_G_2_C_2_T_2_C_2_G_2_G_2_A_1_C_1_	non
pVEC	NH2-KSHAHAQKRIRRRLIILL-COOH	non

## Data Availability

The original contributions presented in this study are included in the article. Further inquiries can be directed to the corresponding author.
